# Sources of perceived social support and emotional difficulties in late adolescence

**DOI:** 10.1093/eurpub/ckac129.441

**Published:** 2022-10-25

**Authors:** V Östberg, SB Låftman, J Raninen

**Affiliations:** 1 Centre for Health Equity Studies, Department of Public Health Sciences, Stockholm, Sweden; 2 Department of Clinical Neuroscience, Karolinska Institutet, Stockholm, Sweden; 3 Centre for Alcohol Policy Research, La Trobe University, Melbourne, Australia

## Abstract

**Background:**

Late adolescence is a life phase when mental health problems are common. Social support is, however, associated with less problems. Social support stems from different sources with the family being the main provider in early life while friends gain in importance during adolescence. Using the Multidimensional Scale of Perceived Social Support (MSPSS), this study investigates the relative importance of different sources of support for emotional difficulties in girls and boys.

**Methods:**

Data was derived from the Swedish population-based cohort study Futura01 (girls: n = 2105, boys: n = 1673). Adolescents in the final year in compulsory school (aged 15-16 years) were sampled in 2017 and followed up in 2019 (aged 17-18 years). Perceived social support was measured at 17-18 years by the MSPSS scale. Emotional difficulties were measured at 15-16 and 17-18 years by the Strengths and Difficulties Questionnaire (SDQ) subscale. Linear regression analysis was used to study associations between support sources and emotional difficulties at 17-18 years, adjusting for all support sources, family type, upper secondary school program as well as prior emotional difficulties.

**Results:**

Among girls, emotional difficulties were associated with perceived support from family (b=-0.22; 95% CI -0.29; -0.15) and friends (b=-0.26; 95% CI -0.35; -0.18) but not significant others (b = 0.00; 95% CI -0.10; 0.11). Among boys, emotional difficulties were associated with support from family (b=-0.10; 95% CI -0.18; -0.02), friends (b=-0.17; 95% CI -0.25; -0.09) and significant others (b=-0.13; 95% CI -0.22; -0.04).

**Conclusions:**

The negative association between perceived social support and emotional difficulties, irrespective of prior emotional difficulties, suggests that support has a protective effect. In late adolescence, support from friends and, to a similar degree, the family seems to matter for mental health. Support from significant others did however not show a protective effect in girls.

**Key messages:**

• Adolescents aged 17-18 years with higher levels of perceived social support report less emotional difficulties.

• Efforts to increase family and friend support are relevant for adolescent mental health.

